# Prediction of Protein–Protein Interaction Sites Based on Stratified Attentional Mechanisms

**DOI:** 10.3389/fgene.2021.784863

**Published:** 2021-11-22

**Authors:** Minli Tang, Longxin Wu, Xinyu Yu, Zhaoqi Chu, Shuting Jin, Juan Liu

**Affiliations:** ^1^ Department of Computer Science and Technology, Xiamen University, Xiamen, China; ^2^ School of Big Data Engineering, Kaili University, Kaili, China; ^3^ Department of Instrumental and Electrical Engineering, School of Aerospace Engineering, Xiamen University, Xiamen, China; ^4^ National Institute for Data Science in Health and Medicine, Xiamen University, Xiamen, China

**Keywords:** protein–protein interaction, multilevel attention mechanism, feature fusion, deep learning, protein features

## Abstract

Proteins are the basic substances that undertake human life activities, and they often perform their biological functions through interactions with other biological macromolecules, such as cell transmission and signal transduction. Predicting the interaction sites between proteins can deepen the understanding of the principle of protein interactions, but traditional experimental methods are time-consuming and labor-intensive. In this study, a new hierarchical attention network structure, named HANPPIS, by adding six effective features of protein sequence, position-specific scoring matrix (PSSM), secondary structure, pre-training vector, hydrophilic, and amino acid position, is proposed to predict protein–protein interaction (PPI) sites. The experiment proved that our model has obtained very effective results, which was better than the existing advanced calculation methods. More importantly, we used the double-layer attention mechanism to improve the interpretability of the model and to a certain extent solved the problem of the “black box” of deep neural networks, which can be used as a reference for location positioning on the biological level.

## Introduction

Proteins participate in various biological processes in organisms. They usually do not play a single role but interact with other biological macromolecules to perform biological functions (Geng et al., 2015). Protein–protein interactions (PPIs) refer to the process in which two or more protein molecules form a protein complex through non-covalent bonds. Protein interactions play an extremely important role in most biochemical functions (Bradford and Westhead, 2005; Nilofer et al., 2020). The identification of protein interaction sites can help researchers understand how proteins perform their biological functions (Ofran and Rost, 2003; Nilofer et al., 2020), and it can also help design new antibacterial drugs (Gainza et al., 2020). Conventional biological experimental methods, such as two-hybrid screening, affinity purification, and mass spectrometry, can be used to identify protein interaction sites (Chung et al., 2007; Gainza et al., 2020). Biological experimental methods have disadvantages of being expensive and time-consuming. Therefore, it is of great value for biologists to develop accurate calculation methods to predict protein interaction sites.

In order to solve the problem concerning expenses, many non-biochemical experimental methods have been developed (Li et al., 2021), and most of the calculation methods are based on machine learning. Zhang and Kurgan (2019) evaluated a large number of functional features that could be used, such as position-specific scoring matrix (PSSM), evolutionary conservation (ECO), and relative solvent accessibility (RSA). In the protein interaction site prediction methods designed by the predecessors, the high score fragment pairs (HSP) as (Li et al., 2021) and the one-hot (Yang et al., 2016; Wang et al., 2019) and amino acid-embedding representations (Zeng et al., 2020) were used to characterize protein sequences as model input features. Wang et al. (2010) proposed a new method for predicting protein interaction sites in hybrids by using the radial basis function neural network (RBFNN) model. This method only used the evolutionary conservation information of the protein and the spatial sequence profile and has achieved good prediction results.

Zhou and Shan (2001) proposed a neural network-based prediction method, taking the sequence distribution of adjacent amino acids and solvent exposure as input. Ofran and Rost (2006) proposed a neural network model for the interaction sites identified from sequence (ISIS), which were trained based on sequence contours and structural features predicted by the sequence. Porollo and Meller (2007) proposed a method named SPPIDER based on the support vector machine, neural network, and linear discriminant analysis, which used 19 features extracted from the sequence. Mizuguchi and Mizuguchi (2010) developed a predictor called PSIVER, which is a naive Bayes classifier based on a position-specific scoring matrix (PSSM), predicting relative solvent accessibility and kernel density estimation. Dhole et al. (2014) proposed a logistic regression classifier LORIS that uses L1 regularization. In addition, Dhole et al. (2015) proposed a new artificial neural network prediction method that used PSSM features, average cumulative hydrophilicity, and predicted relative solvent accessibility to train SPRINGS.

In these studies, a large number of features extracted from protein sequences are used. The commonly used features include evolutionary information and secondary structure (Murakami and Mizuguchi, 2010). In addition to these commonly used features, there are some other physical, chemical, biological, and statistical features, such as the accessible surface area of the protein, protein size, backbone flexibility, and sequence specificity, which have been used for protein interaction site prediction. However, existing methods tend to pay too much attention to protein sequence information, ignoring the characteristics of proteins at the biological level, and most machine learning methods are inexplicable.

In order to solve the above problems, we propose a double-layer attention mechanism prediction model based on graph convolution that uses multidimensional features as input. The main contributions are as follows:

1) For paying more attention to the features at the biological level, we add six effective features of proteins as the input of the model, which can dig out more potential information.
2) The use of the double-layer attention mechanism improves the performance and interpretability of the model and solves the “black box” problem of deep neural networks to a certain extent.

## Methods

### Data

In this experiment, we used three benchmark data sets, namely Dset_186, Dset_72 (Murakami and Mizuguchi, 2010), and Dset_164 (Geng et al., 2015). Dset_186 is constructed from the Protein Data Ban (PDB) database, which is dedicated to the three-dimensional structure of proteins and nucleic acids. Dset_186 is composed of 186 protein sequences, and their sequence homology is less than 25%, and through X-ray crystallography, their resolution is found to be less than 3 Å. The structure of Dset_72 and Dset_164 is the same as that of Dset_186. Dset_72 contains 72 protein sequences, and Dset_164 consists of 164 protein sequences. Therefore, we have a total of 422 different protein sequences. In this study, if an amino acid has an absolute solvent proximity less than 1Å^2^ before and after binding with other proteins, then it is defined as the interaction site; otherwise, it is defined as the non-reciprocal site of action.

Dset_186, Dset_72, and Dset_164 contain 1,923, 5,517 and 6,096 active sites and 16,217, 30,702 and 27,585 non-interactive sites, respectively. Although the protein sequences in the three data sets are not duplicated, the three data sets are from different research groups. So, in order to ensure that the training set and the test set have the same distribution, we integrated the three datasets into a fusion data set. Next, we divided the fused data set into a training set (approximately 80% of the randomly selected protein sequences) and a test set (the remaining 20% of the protein sequences). In the end, we obtained 350 protein sequences in the training set and 70 protein sequences in the test set. Among them, we deleted two protein sequences without defined secondary structure of proteins (DSSP).

### Feature Generation

Feature generation is a key step in the deep learning framework. Excellent features can perfectly represent the various properties of the protein, and features with insufficient expression ability will reduce the accuracy of the deep learning model. In order to better obtain the global features of the protein, we combined six effective features of the protein amino acid encoding, sequence, and structure as input vectors for training. These features include protein sequence, PSSM matrix, secondary structure, pre-training vector, hydrophilicity, and amino acid location.

#### Amino Acid Encoding

***One-hot encoding.*** One-hot encoding is one of the simplest but very effective features, because the original protein sequence can accurately represent each amino acid and its position. Most proteins are composed of 20 different amino acids, so we use 20-dimensional one-hot codes to represent the types of various amino acids in the protein.

#### Sequence Features

***PSSM matrix.*** The evolutionary information in PSSM (Jeong et al., 2011) has been proven to be effective for PPI site prediction. We run the PSI-BLAST algorithm and search NCBI’s non-redundant sequence database with three iterations and a threshold of 0.001 to generate the PSSM matrix. Each amino acid in the protein sequence is encoded as a vector with 20 numbers, which represents the probability of these 20 amino acids appearing at that position.

***Hydrophilic characteristics***. The hydrophilic characteristics of amino acids are determined through experiments, and this characteristic determines the free energy of the transfer of each amino acid. In detail, it is determined by the amount of change in the free energy of amino acids when they move from water to organic solvents. It can be measured by solubility in water and organic solvents. It also contains energetic information about protein interactions and is a very important feature in protein.

#### Structure Features

***Protein secondary structure.*** Secondary structure features are often used in protein prediction. We use the DSSP program to generate secondary structure information. It encodes the structural information of amino acids and uses it to predict protein interaction sites. In this article, we use eight types of secondary structure states (G (3_10-helix), H (α-helix), I (π-helix), B (isolated bridge), E (extended sheet), T (β-turn), S (bend), and other states). Considering that some amino acids have no secondary structure status in the DSSP file, we use a one-hot vector of dimension 9 to encode them. The first eight dimensions indicate the state of each amino acid, and the last dimension indicates whether there is information about the state of the related secondary structure.

***Pre-training vector based on SeqVec.*** In this experiment, we use the pre-training model, that is, SeqVec to obtain the pre-training vector. SeqVec is a protein sequence pre-training model trained using deep unsupervised learning (Villegas-Morcillo et al., 2020). It is based on the model ELMo (Heinzinger et al., 2019) and consists of a character-level convolutional neural network (char-CNN) and two-layer bidirectional long short-term memory (LSTM). The CNN embeds each amino acid in a latent space, generates the corresponding feature vector, and then uses LSTM to model the context of the surrounding amino acids. The model adds two LSTM layers to provide the final context-aware embedding. These embeddings indicate excellent performance in protein classification tasks, such as inferring protein secondary structure, structural category, disordered regions, and cell location. At the level of each amino acid, the predicted secondary structure and the regions with inherent disorder are significantly better than one-hot encoding or the method generated by Word2vec. The generation of protein embedding representations is rapid, and it only takes 0.03 s for SeqVec to generate the evolution information of the target protein. We choose to use SeqVec to represent each amino acid in the sequence as a feature vector with a dimension of 1,024 for subsequent training.

***Residue location characteristics.*** The DeepPPISP model proposed by Zeng et al. (2020) shows that the global information of proteins helps predict protein interaction sites. We use the position information of each residue as the input feature because it provides global position information, and it can also make up for the defect that the attention model cannot capture in position information. The position of the residue in the protein is between 1 and L (protein length). We divide the position by the length of the protein so that a final value between 0 and 1 is obtained and then use this value as the residue position feature and input the model for training.

### Model Structure

The hierarchical attention network (HAN) model (Yang et al., 2016) uses a multilevel attention mechanism to classify documents and has achieved good results. The HAN model has two notable features: 1) a hierarchical thinking to represent documents is used. The document is regarded as composed of sentences, and the sentences are regarded as composed of words and 2) the HAN model applies two attention mechanisms, which are used in documents. At the document level and sentence level, the attention weight of the words is calculated to obtain the representation of the sentence, and then the attention weight of the sentence is calculated to obtain the representation of the document. The abovementioned mechanism enables the HAN to give different sentences and words at different degrees of importance.

**FIGURE 1:**
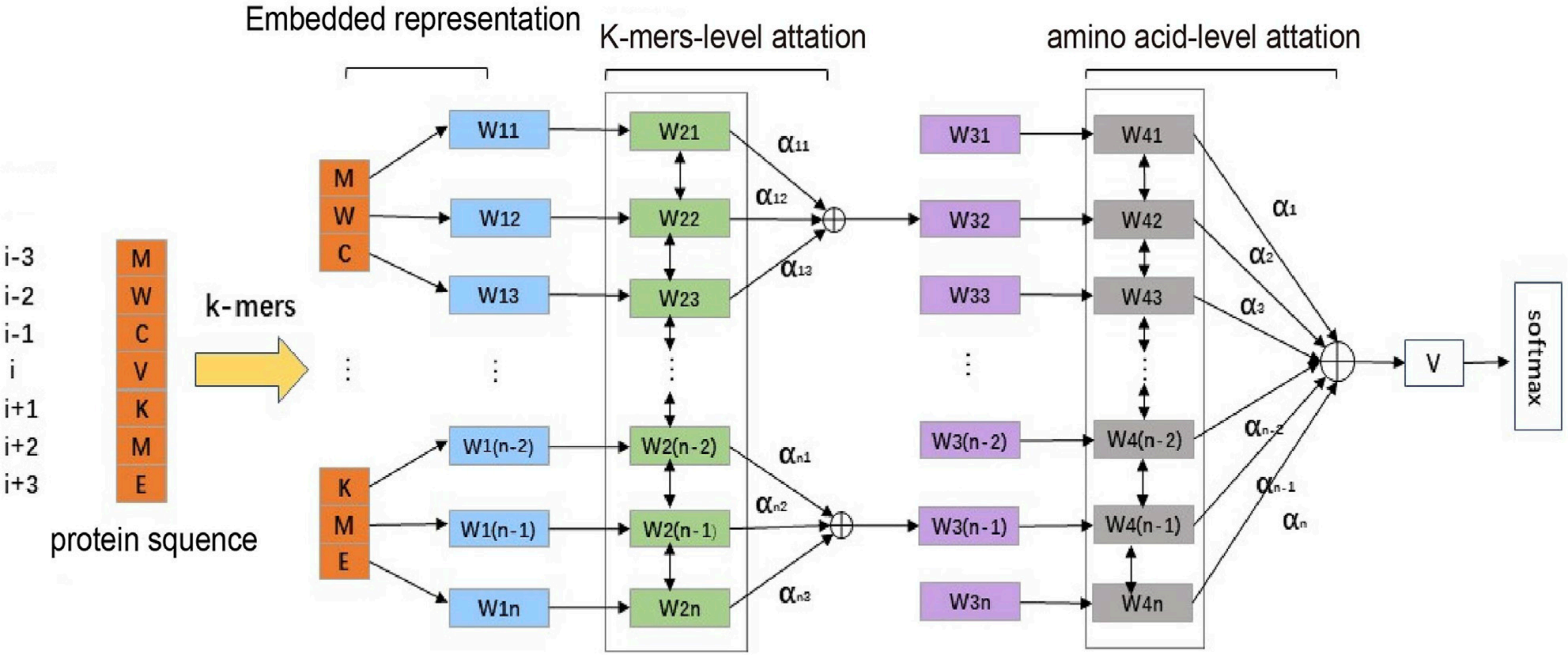
Structure of HANPPIS. It consists of three steps, including embedded representation, amino acid–level attention and K-mers–level attention. We obtain vector representations of protein sequence fragments through multidimensional features. The vector representation of the protein fragment is the input to the first layer of the attention mechanism, and then the vector representation of the protein sequence is obtained through the second layer of attention and finally input to the prediction layer.

Inspired by the HAN, we applied it to the task of predicting PPI sites. The structure of our model is shown in Figure 1. First, we use a sliding window to obtain protein sequence fragments representing protein interaction sites and then divide the fragments into smaller fragments through K-mers. Next, we compare individual amino acids to words in the document. K-mers are analogous to sentences in documents, and the entire protein sequence fragment is analogous to documents. Then, the hierarchical attention model is used for training and prediction, and the final model is obtained. The model obtained in this way can identify the contribution degree of a single amino acid to K-mers and the contribution degree of K-mers to the entire protein sequence fragment. By further analysis, we can deduce which amino acids contribute to the target amino acid as the binding site of protein interaction, what is the specific contribution, and which amino acids may be invalid or can be obtained. We could also know the important characteristics of the amino acids that become the interaction sites of proteins.

In the model, we use a sliding window to integrate the features of the neighboring amino acids. We divide the fixed-length protein sequence into multiple fragments using K-mers and then use the previously introduced method to vectorize the amino acids in the fragments. After obtaining the vector representation of all amino acids, we use the Bi-GRU to encode each amino acid and then use the attention mechanism to calculate the importance of each amino acid for K-mers. Then, we obtain the vector of each K-mer after weighing and summing. We use the same method to encode the K-mer vector, obtain the vector representation of the protein sequence through the attention layer, and finally use Softmax for classification.

**FIGURE 2:**
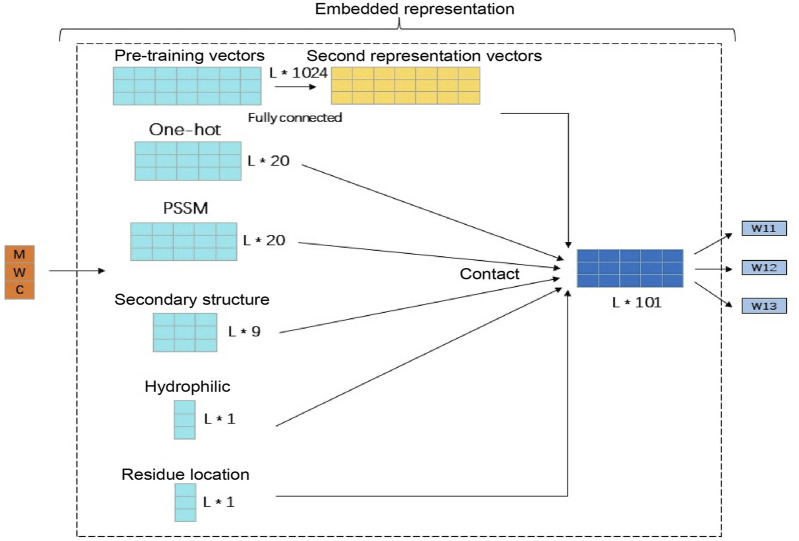
Amino acid feature generation and expression. This figure illustrates the specific details of the amino acid signature generation. Among them, because the pre-training vector feature dimension is too large, a layer of feedforward neural network is used to reduce the dimension to 50 dimensions. Then, the remaining five features are spliced and finally the 101-dimensional amino acid feature vector as the input of the entire model is obtained.

The features we use include the 20-dimensional one-hot amino acid feature, the PSSM matrix feature with the same dimension of 20, the 9-dimensional secondary structure feature, the 1-dimensional hydrophilic feature, the 1-dimensional amino acid position feature, and 1,024-dimensional pre-training vector feature. In order to prevent the pre-training vector feature dimensionality from being too large and affecting the other four features, we used a layer of feedforward neural network to reduce its dimensionality, reducing it to 50 dimensions, and then splicing the other five features to finally get the 101-dimensional amino acid feature representation vector which is used as the input of the entire model. The processing process is shown in Figure 2.

### Model Training Settings

Our deep learning framework is implemented through Keras. The loss function we use is the cross-entropy loss function, which is defined as follows:

$$Loss=-\frac{1}{n}\sum \left[ylog\left(y_{pred}\right)+\left(1-y\right)\text{log}\left(1-y_{pred}\right)\right],$$

where n is the number of all training data, y  is the real label, and $y_{pred}$ is the predicted label.

Our model uses Adam as the optimizer and the following formula to update the weights:

$$\theta_{t+1}=\theta_{t}-\frac{\alpha }{\sqrt{\hat{v_{t}}}+\varepsilon },$$

where $\theta_{t+1}$ is the updated parameter, α is the learning rate, ε  is the constant added to maintain numerical stability, and $\hat{m_{t}}$ and $\hat{v_{t}}$ are the first and second moments after deviation correction, respectively.

In order to extract the contextual sequence features of amino acids at protein interaction sites, we set the sliding window length to 7 and the protein sequence length to 500. Protein sequences longer than 500 will be truncated. For the deep learning model, we set the training batch size to 3, the number of neurons in the LSTM layer and the attention layer in the double-layer attention are both set to 86, and the fully connected layer connected by the pre-training vector has 50 neurons. The positive and negative samples of the training set are not uniformly distributed, so we set the sample weight at about 1:7, which allows the model to pay more attention to the positive samples during training and improve the performance of the model.

## Results and Discussions

### Comparison With the Benchmark Method

To evaluate the performance of HANPPIS in predicting protein interaction sites, we compared HANPPIS with seven competing methods. These six competitive methods all use machine learning or deep learning methods as model training. SPPIDER (Porollo and Meller, 2007) uses an alternative machine learning technology, which combines fingerprints with other sequence and structural information to predict PPI sites. ISIS (Ofran and Rost, 2006) uses a shallow neural network to combine predicted structural features with evolutionary information to predict PPI sites. RF_PPI was developed by Hou et al. (2017). This algorithm uses various protein functions and characteristics and applies it to the random forest algorithm to predict protein interaction sites. PSIVER (Murakami and Mizuguchi, 2010) used sequence features (PSSM matrix and predicted accessibility) and then used a naive Bayes classifier to predict PPI sites. SPRINGS (Dhole et al., 2015) used a shallow neural network algorithm based on evolutionary information, average cumulative hydrophilicity, and predictive relative solvent accessibility to predict PPI sites. In addition, we used graph CNNs to predict PPI sites (GCNPPIS) as a comparative experimental model. The comparison results are shown in Table 1.

**TABLE 1:** Model compares the experimental results on the test set.

Model	Accuracy	Precision	Recall	F1
SPPIDER	0.622	0.209	0.459	0.287
ISIS	0.694	0.211	0.362	0.267
RF_PPI	0.598	0.173	0.512	0.258
PSIVER	0.653	0.253	0.468	0.328
SPRINGS	0.631	0.248	0.598	0.350
GCNPPIS	0.623	0.233	0.395	0.293
HANPPIS	0.631	0.291	0.605	0.393

Table 1 shows the results of HANPPIS and other seven competitive methods on the test set. It is not difficult to find that most of the evaluation indicators measured by HANPPIS are higher than other competitive methods. Although the accuracy rate of HANPPIS is not the highest, other evaluation indicators are higher than competitive methods. Since protein interaction site prediction is an unbalanced learning issue, the ratio of positive and negative data samples is about 1:5.5, so we pay more attention to F1 in the evaluation indicators. Among all existing methods, HANPPIS has the highest F1 value, surpassing existing models.

### Influence of Different Input Features

Obviously, different types of features (original protein sequence, PSSM matrix, secondary structure, hydrophilicity, positional features, pre-training vectors) play different roles in the model. In order to evaluate the importance of each feature, we delete each input feature of HANPPIS separately in the ablation experiment. Specifically, we compared the performance of different models that delete the original protein sequence, that is, PSSM matrix, secondary structure features, hydrophilic features, location features, and pre-training vectors. In order to distinguish between different models, we concluded the following definitions:

1) Model_Dpsf: Delete original protein sequence features
2) Model_Dpm: Delete the PSSM matrix
3) Model_Dssf: Delete secondary structure features
4) Model_Dhf: Delete hydrophilic features
5) Model_Dlf: Delete location features
6) Model_Dptv: Delete the pre-training vector

The results of the ablation experiment are shown in Table 2. The results show that deleting the one-hot feature and the pre-training vector feature has the greatest impact on the model. After deletion, all indicators of the model are reduced at the same time, and the F1 value is as low as 0.319. When deleting several other features, the performance of the model also drops slightly. The experimental results show that comprehensive consideration of these features can obtain more comprehensive protein sequence information, which is helpful to improve the performance of the model and obtain better prediction results.

**TABLE 2:** Results of ablation experiments.

Model	Accuracy	Precision	Recall	F1
Model_Dpsf	0.554	0.237	0.580	0.352
Model_Dpm	0.605	0.269	0.635	0.378
Model_Dssf	0.594	0.263	0.612	0.374
Model_Dhf	0.632	0.282	0.591	0.381
Model_Dlf	0.622	0.295	0.588	0.379
Model_Dptv	0.578	0.275	0.510	0.319
HANPPIS	0.631	0.291	0.605	0.393

### The Effect of Sliding Window Size

In addition to testing different feature inputs, we also studied the impact of different sizes of sliding windows on the model. Specifically, we use sliding windows of different lengths, that is, 7, 9, 11, 13, and 15 to observe the performance of HANPPIS. The results in Table3 show that the model has the highest F1 value when the length of the sliding window is 7.

**TABLE 3:** Effect of the sliding window on the model.

Window size	Accuracy	Precision	Recall	F1
7	0.631	0.291	0.605	0.393
9	0.638	0.298	0.551	0.381
11	0.601	0.281	0.593	0.387
13	0.654	0.300	0.540	0.379
15	0.578	0.275	0.690	0.389

The sliding window has less impact on model performance. It may be because the task is to classify specific amino acids, and the surrounding amino acids are only used as a context to assist. In order to further verify our conjecture, we performed a visual analysis of the attention weight, and the details can be seen in the *Interpretability of the model* section.

### Interpretability of the Model

**FIGURE 3:**
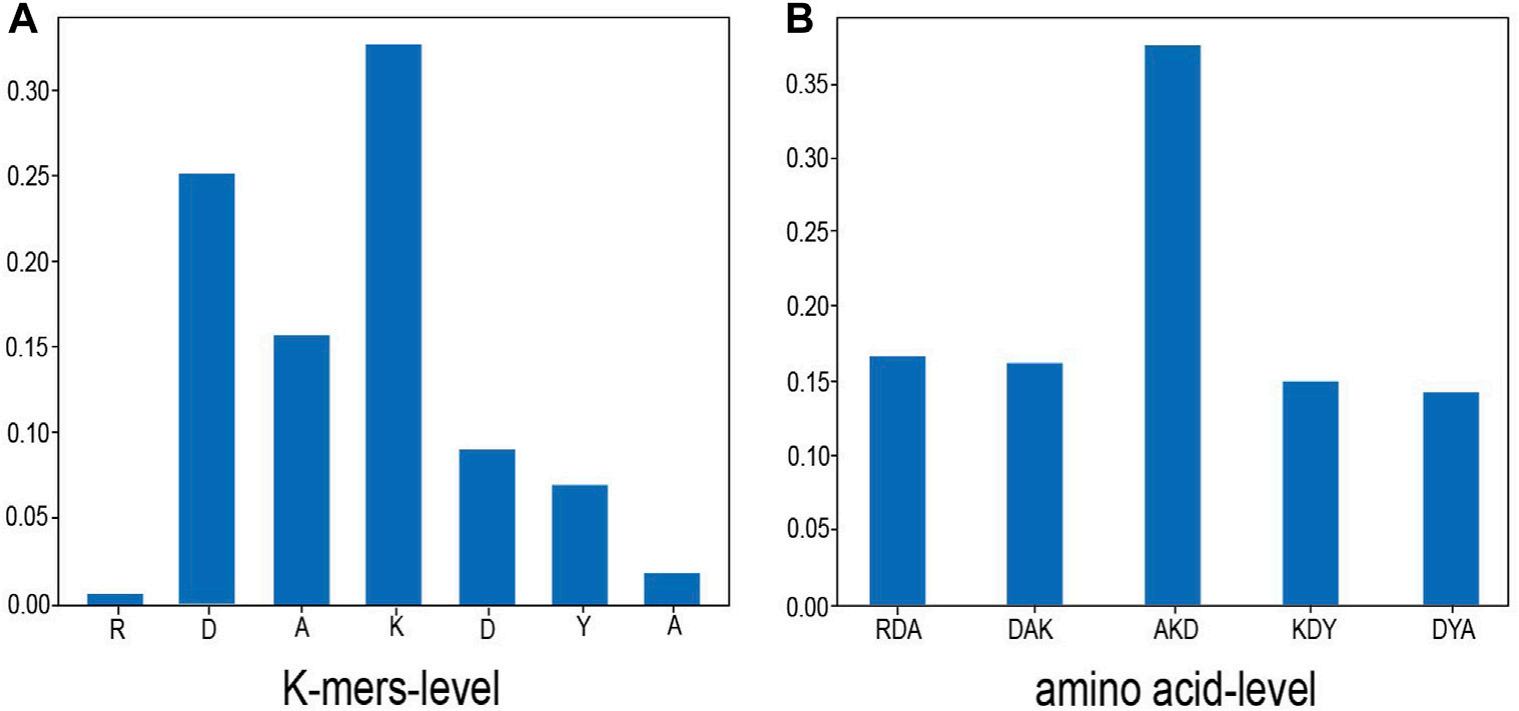
Attention distribution. This figure shows the attention visualization result of one of the samples (from the 135th amino acid of protein 1Z0J_A in Dset186, the sample sequence is “RDAKDYA” and the target amino acid is “K”). **(A)** shows the proportion of K-mers–level attention distribution and **(B)** shows the distribution of amino acid–level attention. As shown in the figure, the center position has the highest proportion of attention, which is also consistent with the task of protein interaction sites.

In order to overcome the common “black box” problem of deep learning and understand the role of contextual amino acids, we randomly selected a sample, which is the 135th amino acid of the protein 1Z0J_A in Dset186. The sample sequence is “RDAKDYA”, and the target amino acid is “K”. We visualized the attention distribution of K-mers–level and amino acid–level to view the contribution of each amino acid or K-mers to protein interaction sites. The experimental results are shown in Figure 3.

As shown in Figure 3, in the K-mers–level attention, it can be seen that the central position of K-mers has the highest attention ratio, and the surrounding K-mers attention weight is less than half of the central position. In the amino acid–level attention, the “K” in the center position also is assigned the highest attention weight. This is consistent with the situation of the protein interaction site task. Because the prediction is whether the central amino acid is the binding site and the surrounding amino acids exist as auxiliary information of the central amino acid, the model’s attention to the center position will be larger, and the proportion of the two sides will pay less attention.

It can be seen from Figure 3B that the attention weights of amino acids “R” and “A” are both less than 0.05, which is probably the reason why the sliding window does not have a high degree of influence on the model because the farther away from the center, the lower the weight of the amino acid. Therefore, even if the sliding window is enlarged, it has little effect on the amino acids in the central position.

In general, we verified that HANPPIS is suitable for discovering important patterns in protein sequences, and the attention mechanism can understand its relationship in context, which greatly increases the interpretability of the model.

## Conclusion

The accurate prediction of protein interaction sites can promote the understanding of protein biological functions. In this article, we propose a deep learning framework HANPPIS to predict protein interaction sites at the amino acid level. The difference between HANPPIS and other existing methods is that the model uses hierarchical attention combined with neural networks to predict protein interaction sites. HANPPIS captures global sequence features through Bi-GRU, so that it can easily simulate the relationship between the target amino acid and the entire protein sequence. After Bi-GRU processing, the attention layer is used to let the model assign higher weights to the parts that need attention, and further follow-up results can be obtained. The experiment was repeated twice to generate attention weights for amino acids and K-mers and finally classify and output them through Softmax. The results show that HANPPIS basically surpasses the existing competitive methods in the task of predicting protein interaction sites. Sequence-based protein interaction site prediction is still a challenging problem, and one of the reasons is that there are no unique attributes in the sequence to directly analyze the protein sequence. But in this study, we showed that hierarchical attention can be used for protein interaction site prediction, and more important parts of disordered protein sequences can be found. The multiple experimental results also demonstrated the crucial role of attention mechanism that can increase the interpretability of the model and provided the possibility and direction for further exploration of the mystery of proteins.

But our method has some limitations, such as HANPPIS requires the multidimensional features of the protein as input. Obviously, these features may be missing in some data sets. In addition, the samples for testing attention visualization in the experiment are not enough. In future studies, we would be committed to use fewer features to obtain better performance and improve the interpretability of the model.

## Data Availability

The original contributions presented in the study are included in the article/Supplementary Material; further inquiries can be directed to the corresponding authors.
